# Book Review: An adventurous life of Victor Ivanovich Motschulsky, described by himself: Compiled by V.A. Krivokhatsky, Edited by K.G. Mikhailov

**DOI:** 10.3897/zookeys.371.7028

**Published:** 2014-01-17

**Authors:** Kiril Mikhailov, Sergei Golovatch

**Affiliations:** 1 MGU Musum of Zoology, Bolshaya Nikitska St. d.6, p.c. 125009, Moscow, Russia; 2 Russian Academy of Sciences, Institute for Problems of Ecology and Evolution, Leninsky pr. 33, p.c. 119071, Moscow, Russia

It is difficult to review the book one is editing. One sees the flaws, having already received comments from colleagues, zoologists and historians of science. But V.I. Motschulsky is such a compelling yet little-known personality that we find it our duty to review the book even though one of us is its editor.

Among entomologists, rumors abound that Motschulsky, an outstanding specialist in insect systematics as well as an avid collector, stole beetle specimens from famous collections and argued with his European colleagues. He had a difficult personality. It is no accident that Walter Horn called him “the infernal entomologist.”

History and natural history are intertwined in the book, which consists in large part of memoirs recorded in Russian and German, and in part of reverse translations of Motschulsky’s articles published in the 1850s–60s mostly in French. The first part is, first and foremost, a history, while the second is largely natural history. (Let us not forget that “biology”, while coined as a term, was not widely used until the late 19^th^ century). The translation from German of the biographical and highly biased essay by W. Horn, which was written in the 1920s, is placed in the end of the book.

V.I. Motschulsky was born in 1810. A military man, he participated in the suppression of the Polish uprising, where he was wounded and lost hearing in the left ear. Later, he served in the Caucasus, the so-called Kyrgyz steppe, East Siberia, and Orenburg. The biographical account ends in 1841, followed by reverse translations of entomological expeditions to the outskirts of St. Petersburg as well as abroad to Europe and America in 1852-55. There remains no account of the period from 1841 to 1851 and from 1856 on. All that remains are headings: “Military service in Chuguyev,” “Travels in the Crimea,” “Travels abroad. Egypt,” “Paris. London. Dalmatia. Montenegro,” “My survey on the gnawing of the French army’s lead bullets by the grub *Urocerus juvenicus* in the Crimea”, etc. In 1863, Motschulsky permanently relocated to the Crimea, was very ill in the last years of his life and died in 1871.

While reading his memoirs, one is stunned by the breadth of Motschulsky’s interests as well as his integrity and patriotism. Motschulsky was a direct participant in such events as the repression of the Polish uprising in 1830-31 and the conquest of the Caucasus and Central Asia. He witnessed the horrific flooding of Kronstadt in November 1824. The Decembrists’ uprising took place when he was a youth and the War of 1812 while he was a baby. In one way or another, all these and other events are reflected in his memoirs. Motschulsky’s book offers a few novel historical facts and interpretations. Apparently, Empress Catherine II was murdered: Lady Perekusikhina served her poisoned chocolate (p. 20). The book recounts the story of a man who impersonated the murdered Emperor Paul (pp. 21, 164). Halfway through the book there appears an authoritative confirmation that Count Araktschejew was, in fact, murdered after stealing the signed letterhead of the late Emperor Alexander I and forging 13 royal decrees (p. 105). Motschulsky purportedly heard this last shocking story directly from Count P.A. Kleinmichel: the book marks its first appearance in scientific literature. Other anecdotes are interspersed among the memoirs. To his credit, Motschulsky had a sober view of the tsarist administration. “Emperor Nicholas exclusively favored the military, whereas members of other professions had to make do elsewise.” (p. 23). “And, given that everyone must serve in the military, and that all young men are groomed exclusively for military service, their remaining talents are stunted. This is truly unfortunate for the empire, because as a result, the landed gentry is weakened, estates are abandoned by their owners and fall into disrepair, families grow destitute, trade is chilled outside of large urban centers, and the country as a whole grows poor.” (p. 23). This is what came of the incompetently run Polish campaign and intrigues and thievery in the Caucasus. It is abundantly clear that the principles and methods of governance in Russia have not changed much since the reign of Nicholas I.

It appears that Motschulsky’s career in the military was not very successful, but he did not aim to succeed there. “On one hand, it instilled in me distaste for the unlawful and on the other, honesty, contrary to which I could not act and due to which I often got in trouble given my volatile temper.” (p. 18). For example, “on two occasions, I was demoted due to my temper: the first time, I called our French teacher a scoundrel and the second time, I could not stop laughing at a demented duty officer.” (p. 22). While stationed in the Caucasus, Motschulsky successfully submitted to his superiors a report on “the Persian and Afghan question” and received recognition for it. As a result, he was to be dispatched to Persia, but the plan fell through because of his poor health. Instead, he was allowed to go on leave “abroad” even though at that time, trips abroad were largely discouraged. On his return in 1836, Motschulsky was offered to continue serving in St. Petersburg, but he chose to return to the Caucasus. Notwithstanding his heroism and professional acclaim, he was ultimately forced out. Later, after his work in the so-called Kyrgyz steppe and East Siberia, the following happened. “I wanted to join the diplomatic corps, to make use of my knowledge of the East, but the Ministry of Foreign Affairs dragged its feet, delaying for many months and many questionable reasons. Tired of waiting, I decided to accept a position as special envoy for the Committee and Commission for the Construction of the Moscow Railway. Thus, all I had assembled and studied over the course of many years bore no fruit.” (p. 169). And, by way of a bitter recap, “After all, in this country, an educated and honest man can find few ways to earn a living.” (p. 22).

Motschulsky’s luck and courage – and even, to a degree, his recklessness – are astounding. Here is an anecdote from the Polish campaign: “A grenade exploded under my horse, and as a result I was thrown in the air but not injured. The horse itself received a mild shrapnel wound. Because we had not eaten anything in two days, one of our Cossacks brought me a pot of beef from camp. My fellow soldiers and I rode off a distance, so as to enjoy the delicacy without running the risk of being shot. We got off our horses, set the pot down on the ground, and sat on the grass, eagerly awaiting a chance to partake of the beef. I reached out with a fork for a piece of beef when we were all covered with a cloud of dust and lost consciousness. The pot and fork disappeared – they were smashed by a ricocheted cannonball. Our food was scattered everywhere in miniscule bits. It appears that we had picked a poor place for a meal.” (p. 40). And here is an account of the Swiss Alps: “When crossing the snowy peaks of Appenzell, we had to walk on very narrow paths, where snow that had melted in the June heat frequently slid out from under our feet. Once, we started slipping downhill – I fell on my back and with terrifying speed began to slide down. But I kept my wits about me and tried to stop my descent, using an iron pick of the kind that every traveler carries. This I succeeded in doing a few feet before the edge of a cliff. Another time, when I slipped and fell in the same area, I was sliding so fast, I could not see anything and everything turned black, when I felt a terrifying jolt resonate through my body. I stopped, my feet pressed against a ridge of rocks and ice that had formed at the edge of a cliff where goats had traveled in the fall – just a couple of feet from a drop of over 250 feet.” (p. 86).

Motschulsky’s descriptions of nature and people are full of incisive observations. He describes Caucasian mineral waters; Prince Sevardzelidze, a man of legendary strength; the bazaars of Tiflis; the Georgian temperament; oil spills in Dagestan; Caucasian mountain peaks; and jackals who stole the boots he had treated with cured pig fat, as he slept outside, fearing the fleas that abounded indoors.

At the time of Motschulsky’s early travels, oil was not yet widely used. “In the 1820s, near Sary island in the Caspian Sea, a new island had formed and Mr. Ménétriés [a renowned naturalist from St. Petersburg – *K.M.* & *S.G.*] traveled by boat to inspect it. One of the sailors carelessly lit a pipe, a spark fell, and the island went up in flames. Ménétriés and his men barely managed to save the boat, but the sea was covered in oil and also lit up. They barely made it out, rowing as fast as they could.” (p. 64). Describing Astrakhan and its vicinity, Motschulsky justly notes, “Extracting oil from the islands and coastal regions of the Caspian Sea alone could form a substantial basis for trade if petroleum were adopted for illuminating streets and houses, where it may well replace gas and, even more so, other oils.” (p. 147).

During his travels abroad, Motschulsky was interested not only in beetles, but also museums, architecture (cathedrals, monuments, tombs), and the memorial museum of Andreas Hofer, a famous partisan from Tyrol, who fought against Napoleon’s invasion. In the museum, which was once Hofer’s residence, “visitors can see his trousers and even snip off a piece as a souvenir.” (p. 90–91).

Motschulsky engaged in occasional discussions on economics, concerned about a well-run economy. Here is what he writes about the fishing trades in Astrakhan: “To strengthen the fishing trades, we must clean Volga’s polluted estuary so that fish may enter the river from the sea, which is presently difficult if not impossible. It is noticeable how sturgeon and beluga get stranded in the shallows and cannot get out: it is hardly surprising that many fish return to the sea and seek out other estuaries to fulfill their natural cycles. It also bears noting that now, fishermen leave on the shore small fish not intended for trade, whereas previously, they released it back into the sea, thus preserving the young. At present, however, one can smell millions of rotting fish, dying for no good reason around fishing rafts and boats.” (p. 147).

But Motschulsky’s primary passion was, of course, beetles and, to a lesser degree, other insects. He collected them on every suitable occasion – in the Caucasus, in the outskirts of St. Petersburg, in America. The beetles he caught he literally stuffed in his pockets (in pill boxes) and later emptied out onto sticky sheets covered with gum arabic. He then divided the sheets into segments and mounted them on entomological pins. These sheets are still part of his beetle collection. Motschulsky actively communicated with the leading entomologists of his day, frequently arguing with them and engaging in disputes. Motschulsky’s collection was bequeathed to the Moscow Society of Naturalists, where it was abandoned. It was not re-discovered until the early 20^th^ century and is currently housed in Moscow University’s Zoological Museum. It has partly been re-examined and many beetles have been reclassified according to modern standards. But the boxes with other insects, myriapods and crustaceans are in very poor shape. They have been ravaged by dermestid beetle larvae, an entomologist’s worst enemy. The small collection of spiders has been lost altogether.

In fact, beetles saved Motschulsky’s life when he was sent on a reconnaissance mission together with several locals loyal to the Russian government. Pretending to be deaf and dumb, he was captured by highlanders in the Caucasus in 1838. He was “made” when his captors noticed that his hood was stitched in silk, as was customary in Tiflis, and not in wool, as in the mountains. “Suddenly, we were surrounded by highlanders, who took our horses and weapons. They put each of us in a separate hut under guard.” (p. 135). “As soon as I realized what danger I was in, I did not fail to make friends. The first was this woman [a highlander’s wife who was told to guard Motschulsky – *K.M.* & *S.G.*]. Dinnertime was approaching, and I was helping her with the preparations, fixing the fire, making flatbread, and otherwise assisting with small chores – all was done as in accordance with the highlanders’ customs. In the meantime, I slipped into the fire a pencil I was carrying in my pocket, which, if found, would undoubtedly have costed me my life.” (p. 136). The prisoner was led into a clearing between cliffs and a precipice in the hopes that he would start talking to his travel companions and reveal his purpose. “Knowing quite well that our enemies were watching us, I remained true to my assumed role and, paying no attention to my travel companions, I lay down on the ground and started playing with rocks, putting them in my mouth and spitting them out into the abyss. In the meantime, I was shredding into bits the piece of paper I was carrying in my pocket, chewing and swallowing the bits. Thus, I got rid of a dangerous and incriminating item which contained notes from my travels. [A while later,] the highlanders came and searched my pockets, where they found a few crushed insects I had gathered along the way, the discovery of which caused uproarious laughter, and which they carefully returned to my pocket.” (p. 136).

The chapter titled “1833. Lithuania. Magnetism” stands out somewhat. In it, Motschulsky encounters magnetism, or lunacy, in his cousin Louisa. This condition, together with Louisa’s youth – Motschulsky planned to marry her upon her recovery – greatly appealed to the scientist. But the marriage did not take place, most likely due to his numerous travels and growing interest in beetles. The chapter remains unfinished, ending literally mid-word.

The manuscript containing Motschulsky’s memoirs was illustrated with landscapes and drawings of beetles and other arthropods, but unfortunately, far from all of these illustrations have survived. The book contains all that could be located. Certain peculiarities of Motschulsky’s language should also be kept in mind, for clarity’s sake. For example, he calls robbers predators and beggars the bedraggled.

In the introduction, the compiler of the book compares Motschulsky to Paganel, but this fictional geographer and naturalist had not yet been created by Jules Verne and came into existence somewhat later, in 1859. A more accurate comparison might be made to Cousin Benedict, an entomologist, and also a creation of Jules Verne.

Despite prolonged – even drawn out – preparations, the book has not been sufficiently edited, and typos have crept in. There is also a noticeable dearth of reference tools: footnotes, index, and list of Latin titles. For example, Motschulsky writes that Lake Baikal supports “a marvelous fish, as large as a ruff, and consisting entirely of fat.” (p. 167). There is no further commentary. Meanwhile, he is apparently speaking of the big Baikal oilfish (Y.V. Tchaikovsky, personal comment). Many names and geographic locations are given multiple spellings, which somewhat interferes with reading the book. Regretfully, Latin names are also sloppily written.

It is difficult to describe in a book review our awe and delight with this wonderful book. The author’s language is perfect, albeit somewhat obsolete Russian, while his views are almost modern. We recommend that you read this book for yourself. Orders from abroad can be made through Pensoft Publishers, <www.pensoft.net>.

Far from all biographic materials of V.I. Motschulsky have been made available to scholars. Research remains to be done in military archives, including his personal file and report on “the Eastern question”. As far as we know, Motschulsky’s business correspondence may still be found in various civil archives in Moscow and St. Petersburg. But an academic biography of our hero is a task for the future.

Moscow & St. Petersburg, KMK Scientific Press, 2013. “The Sphere of Eurasia” series, hardbound, 261 pages. ISBN 978-5-87317-921-3. In Russian.

## References

[B1] Krivokhatsky VA (2013) An adventurous life of Victor Ivanovich Motschulsky, described by himself. KMK Scientific Press, Moscow & St. Petersburg, 261 pp. [in Russian]

